# The use of antibiotics during pregnancy: A cross‐sectional study of knowledge, attitude, and practices among antenatal care attendees in Northern Ghana

**DOI:** 10.1002/hsr2.2111

**Published:** 2024-05-21

**Authors:** Ezekiel K. Vicar, Williams Walana, Rosemond A. Fordjour, Christiana Benneh, Rosemond E. Bentil, Gifty M. Wuffelle, Emmanuel K. Osabutey, Gilbert Nachinab, Mauvina Obeng‐Bempong

**Affiliations:** ^1^ Department of Clinical Microbiology University for Development Studies Tamale Ghana; ^2^ Department of Midwifery and Women's Health, School of Nursing and Midwifery University for Development Studies Tamale Ghana; ^3^ Department of Human Anatomy University for Development Studies Tamale Ghana; ^4^ Department of General Nursing, School of Nursing and Midwifery University for Development Studies Tamale Ghana; ^5^ Department of Pediatrics and Child Health Tamale Teaching Hospital Tamale Ghana

**Keywords:** antibiotics, Ghana, knowledge, pregnancy, self‐medication

## Abstract

**Background and aims:**

The promotion of rational use of antibiotics among pregnant women is eminent not only for the risk of teratogenicity in the developing fetus but also the risk of drug resistance with its concomitant high cost of health care. Studies on antibiotic self‐medication among pregnant women in Northern Ghana are rare. Improving the knowledge and awareness among the vulnerable groups about the appropriate use of antibiotics can help in limiting the antibiotic resistance menace. We, therefore, conducted this study to assess the knowledge, attitude, and practice (KAP) toward antibiotic use among pregnant women attending an antenatal clinic at a primary health care in Tolon, Northern Region, Ghana.

**Method:**

We conducted a cross‐sectional study using an interviewer‐administered questionnaire to assess the KAP of 702 pregnant women on antibiotic use. This study was conducted in the Tolon Health Center (THC) from March 2021 and ended in October 2021.

**Results:**

In this study, 55.6% of pregnant women had good knowledge and 45.3% of them had engaged in self‐medication with antibiotics while pregnant. There were statistically significant associations between participants' background and obstetric characteristics and knowledge of antibiotic use and antibiotic resistance, except for age, marital status, and parity. Also, there was a significant association between pregnant women's knowledge and self‐medication or over‐the‐counter purchase of antibiotics.

**Conclusion:**

We concluded that higher education level, monthly income, good practice, and good knowledge were significantly associated with a reduced likelihood of self‐medication with antibiotics. A well‐structured education that could be easily accepted and understood by pregnant women on the risks of antibiotic self‐medication should be included in the routine education at the antenatal clinics.

## 1 INTRODUCTION

Antibiotic resistance has become a worldwide health challenge associated with the rise in antibiotic prescription and consumption worldwide.^1^ This challenge has a serious consequence on humans, as diseases that were easily treatable will be difficult to treat.^2^ Among many, self‐medication is a major contributor to the development of human pathogen resistance to antibiotic drugs.^2^, ^3^ Pregnant women are one of the vulnerable population groups who practice self‐medication frequently and repeatedly for the prevention of abortion and treatment of pregnancy‐related morbidities.^4^ During pregnancy, many physiological and hormonal changes occur, which make these women susceptible to infection,^5^ which if not managed timely and well, may lead to some complications that may be fatal to the mother or fetus.^6^, ^7^ To bring quick relief from these pregnancy‐related symptoms, pregnant women may resort to using medicines. Because of inefficient health care system and easy access to medicine from several channels, many pregnant women in developing countries easily practice self‐medication.^8^, ^9^, ^10^

Self‐medication during pregnancy presents a serious maternal and fetal health threat that cannot be overemphasized.^11^, ^12^ It may lead to fetal toxicity, low birth weight, preterm delivery, and also other teratogenic effects.^10^, ^13^ Sub‐Saharan Africa accounted for ~66% of the estimated global maternal deaths in 2017.^14^ Many studies have indicated high rates of self‐medication during pregnancy,^4^, ^9^, ^15^, ^16^, ^17^, ^18^, ^19^ with self‐medication with antibiotics among pregnant women reported as 1.9% in Tanzania^16^ and 9.6% in Nigeria.^19^ However, a study in Ghana reported that the prevalence of antibiotic exposure among pregnant women increased from 54.8% in 2013 to 77.8% in 2015.^12^ Antibiotic exposures during pregnancy have been associated with both short‐term (e.g., congenital abnormalities) and long‐term effects (e.g., changes in the gut microbiome, asthma, atopic dermatitis) in the newborn. However, it is estimated that only 10% of medications have sufficient data related to safe and effective use by pregnant women.^20^ Widespread irrational use of antibiotics and ignorance of people about the need to complete the course of antibiotics, their side effects, standard acceptable dosage limits, and antibiotic overdose issues can lead to microbial resistance issues.^2^

The promotion of rational antibiotic use is imminent due to the risk of drug resistance with its concomitant high cost of health care and also the risk of teratogenicity in the developing fetus.^12^ Even though there are many potential dangers associated with antibiotic self‐medication during pregnancy,^21^, ^22^ many pregnant women are oblivious of them.^17^ Improving the knowledge and awareness among the nonmedical population about the usage of antibiotics can help in limiting the antibiotic resistance menace.^2^, ^3^

Report on pregnant women's knowledge, attitudes, and antibiotic use is rare in Ghana, especially in the Northern Region. To speed up the antibiotic stewardship campaign in Northern Ghana and implementation of appropriate interventions, a potential knowledge gap concerning antibiotic use in pregnancy and associated factors must be identified. We, therefore, conducted this study to assess the knowledge, attitude, and practice (KAP) toward antibiotic use among pregnant women attending an antenatal clinic at a primary health care in Tolon, Northern region, Ghana.

## 2 METHOD

### 2.1 Study design and setting

This cross‐sectional study conducted among antenatal attendees at the Tolon Health Center (THC) within the Northern Region of Ghana. This was conducted between May 2021 to October 2021.

The district lies between latitudes 9° 15" and 10° 002′ North and Longitudes 0° 53′ and 1° 25′ West. Tolon has a population of 118,101, with 58,512 being males and 59,589 females.^23^ The annual population change is 4.6% and a population density of 90.06/km^2^.^23^

### 2.2 Study population and eligibility criteria

The study participants were all pregnant women receiving antenatal care (ANC) at THC during the study period. Only pregnant women who gave informed consent were included in the studies. For those below 18 years, their legal guardian or parent consented for them.

### 2.3 Sample size calculation

We used the Yamane's formula to determine the minimum number of participants required for the study. The formula stated that *n* = *N*/1 + *Ne* ^2^; where *n* is the sample size required, N is the known population size, e represents the error (0.05) at a confidence level of 95%. Using a population size of 118,101, the minimum number of participants required for the study was 399.

### 2.4 Sampling methods

We used a simple random technique to select study participants. The facility was visited twice in a week. For each day of visit, a maximum of 15 pregnant women were recruited for the study. In all, we recruited 702 pregnant women over the 6‐month period.

### 2.5 Data collection instrument

We used an interviewer‐administered questionnaire, which was adapted from other studies,^15^, ^24^ to establish the KAPs of pregnant women regarding antibiotic use. The questionnaire constitutes a section for socio‐demographic and obstetric characteristics, KAP. The internal consistency of questions for knowledge sections that were scored was assessed and Cronbach's *α* value of 0.80 was obtained.

Three trained midwifery students with the supervision of a medical officer administered the questionnaires. The questionnaires were pretested before being used. Antenatal attendees who consented by signing or thumbprinting or through their guardian were recruited into the study. The questionnaire was translated to the respondent's local dialect by a language competent midwife when necessary.

### 2.6 Variables

The variables of interest socio‐demographic and obstetric history, KAP regarding antibiotic use.

The main outcomes of interest were knowledge score, desired attitude, and desired practice of antibiotic use. Knowledge score was determined as the number of questions answered correctly out of the total questions. Knowledge score above the mean knowledge score was considered as good knowledge.^15^ For attitude and practice, we calculated the total desired answers and reported them in frequencies. Purchase and use of antibiotics without prescription was considered as self‐medication.

Socio‐demographic and obstetric history, knowledge, and attitude were tested as the main predictors of antibiotic use among the pregnant women.

### 2.7 Data analysis

First, the data entry team checked for the completeness of the questionnaire. Incomplete questionnaires were excluded from analysis. Data were then entered into Microsoft Excel 2019, cleaned, and exported to SPSS version 26 for analysis. For descriptive statistics, frequencies and percentages were used. Logistic regression was used to detect predictors of antibiotic self‐medication and odds ratio, 95% confidence interval (CI), and *p* = 0.05 were used.

### 2.8 Ethical considerations

Permission for the study was obtained from the administrator of the facility. Ethical approval was obtained from University for Development Studies Institutional Review Board with reference UDS/RB/0006/21. The interviews were conducted in privacy and all information was kept confidential.

## 3 RESULTS

### 3.1 Demographic and obstetrics characteristics of participants

As seen in Table 1, participants' age ranged from 16 to 45 years with a mean age of 25.2 ± 3.81 years. Most (54.7%) of the participants were from the age of 21 to 30 years and 660 (94.0%) were married. In terms of monthly income, 61.4% earned <Gh¢500.00 with 12.8% earning over Gh¢2000.00. Among the 702 pregnant women, 24.8% had no formal education and 29.9%, 32.5%, and 12.8% had completed primary, secondary, and tertiary school education, respectively.

**Table 1:** Analysis of knowledge level by background and obstetrics characteristics, attitude, and practice.

Characteristics variable			Good knowledge		Poor knowledge			
	Total	*N* (%)	*n*	(%)	*n*	(%)	*χ* ^2^, df	*p*
Age (years)								
16–20	102	(14.5)	60	(8.5)	42	(6.0)	4.846, 5	0.435
21–25	168	(23.9)	90	(12.8)	78	(11.1)		
26–30	216	(30.8)	120	(17.1)	96	(13.7)		
31–35	90	(12.8)	48	(6.8)	42	(6.0)		
36–40	102	(14.5)	54	(7.7)	48	(6.8)		
≥41	24	(3.4)	18	(2.6)	6	(0.9)		
Marital status								
Not married	42	(6.0)	18	(2.6)	24	(3.4)		0.109
Married	660	(94.0)	372	(53.0)	288	(41.0)		
Educational level								
None	174	(24.8)	18	(2.6)	156	(22.2)	196.8, 3	<0.001
Primary	210	(29.9)	138	(19.7)	72	(10.3)		
Secondary	228	(32.5)	162	(23.1)	66	(9.4)		
Tertiary	90	(12.8)	72	(10.3)	18	(2.6)		
Monthly income								
Less than 500	276	(39.3)	84	(12.0)	192	(27.4)	135.9, 4	<0.001
500–999	150	(21.4)	90	(12.8)	60	(8.5)		
1000–1499	72	(10.3)	48	(6.8)	24	(3.4)		
1500–1999	114	(16.2)	90	(12.8)	24	(3.4)		
≥2000	90	(12.8)	78	(11.1)	12	(1.7)		
Duration of pregnancy								
First trimester	108	(15.4)	66	(9.4)	42	(6.0)	2.411, 2	0.300
Second trimester	318	(45.3)	168	(23.9)	150	(21.4)		
Third trimester	276	(39.3)	156	(22.2)	120	(17.1)		
Gravidity								
First	126	(17.9)	62	(8.8)	64	(9.1)	5.191, 4	0.261
Second	162	(23.1)	84	(12.0)	78	(11.1)		
Third	180	(25.6)	106	(15.1)	74	(10.5)		
Fourth	156	(22.2)	90	(12.8)	66	(9.4)		
Fifth or more	78	(11.1)	48	(6.8)	30	(4.3)		
Parity								
Nulliparous	138	(19.7)	78	(11.1)	60	(8.5)	3.998, 3	0.262
Primiparous	198	(28.2)	108	(15.4)	90	(12.8)		
Multiparous	342	(48.7)	186	(26.5)	156	(22.2)		
Grand multiparous	24	(3.4)	18	(2.6)	6	(0.9)		
Ever lost pregnancy								
No	438	(62.4)	276	(39.3)	162	(23.1)		<0.001
Yes	264	(37.6)	114	(16.2)	150	(21.4)		
Number of pregnancies lost (*n* = 264)								
One	216	(30.8)	84	(12.0)	132	(18.8)	10.10, 2	0.006
Two	42	(6.0)	18	(2.6)	24	(3.4)		
Three	6	(0.9)	6	(0.9)	0	(0.0)		
Pregnant women should not accept antibiotics from family to treat an infection								
Disagree	300	(42.7)	156	(22.2)	144	(20.5)	2.833, 2	0.243
Agree	312	(44.4)	180	(25.6)	132	(18.8)		
Neutral	90	(12.8)	54	(7.7)	36	(5.1)		
Pregnant women must consult a doctor before taking antibiotic								
Disagree	60	(8.5)	36	(5.1)	24	(3.4)	0.5979, 2	0.742
Agree	600	(85.5)	330	(47.0)	270	(38.5)		
Neutral	42	(6.0)	24	(3.4)	18	(2.6)		
Pregnant women should not purchase antibiotics over the counter								
Disagree	108	(15.4)	54	(7.7)	54	(7.7)	13.87, 2	0.001
Agree	528	(75.2)	312	(44.4)	216	(30.8)		
Neutral	66	(9.4)	24	(3.4)	42	(6.0)		
Do you complete the course of regimen of antibiotic treatment								
No	396	(56.4)	222	(31.6)	174	(24.8)		0.760
Yes	306	(43.6)	168	(23.9)	138	(19.7)		
Do you take more than the prescribed amount of antibiotic when you do feel well								
No	672	(95.7)	366	(52.1)	306	(43.6)		0.008
Yes	30	(4.3)	24	(3.4)	6	(0.9)		
Do you keep non‐prescribed antibiotics at home in case you would need it								
No	384	(54.7)	228	(32.5)	156	(22.2)		0.027
Yes	318	(45.3)	162	(23.1)	156	(22.2)		
Do you purchase antibiotics from over‐the ‐counter for an infection?								
No	384	(54.7)	294	(41.9)	90	(12.8)		<0.001
Yes	318	(45.3)	96	(13.7)	222	(31.6)		

Also, in the study were 138(19.7%) nulliparous, 198(28.2%) were primiparous, 342(48.7%) multiparous, and 24(3.4%) grand multiparous women. Three hundred and eighteen (45.3%) of the pregnant women were in their second trimester with an average number of gravidae of 2.85 ± 1. 264 and 264 (37.6%) had lost a pregnancy.

### 3.2 Knowledge of antibiotic use among pregnant women

**Figure 1:**
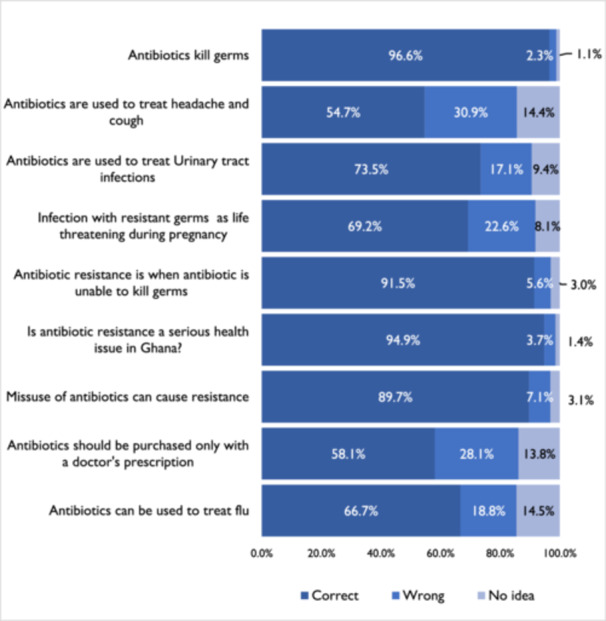
Percentage of responses from all respondents to statements to determine knowledge towards antibiotic use.

We used nine questions to assess participants' knowledge of antibiotic usage during pregnancy and antibiotic resistance. The mean knowledge score was 6.5 ± 1.2., which was the minimum score for good knowledge. Three hundred and ninety (55.6%) had good knowledge. More than half of the participants, 54.7% and 66.7% knew that antibiotics are not used to treat headaches and cough, and antibiotics can be used to treat flu, respectively. Also, 91.5% knew that antibiotic resistance is when an antibiotic is unable to kill germs and 94.9% knew that resistance is a serious health issue (Figure 1).

There were statistically significant associations between participants' background and obstetric characteristics and knowledge of antibiotic use and antibiotic resistance, except for age (*p* = 0.435), marital status (*p* = 0.109), and parity (*p* = 0.262). Also, there was a significant association between pregnant women's knowledge and self‐medication or over‐the‐counter purchase of antibiotics (Table 1 and Figure 1).

### 3.3 Attitudes of pregnant women towards antibiotic use

**Figure 2:**
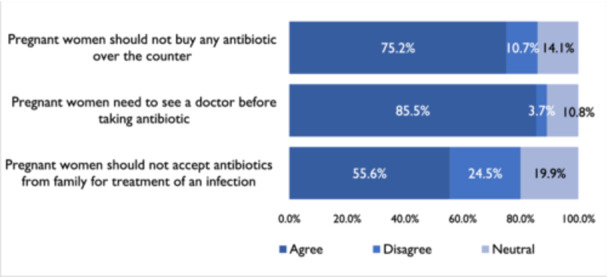
Percentage of responses from all respondents to statements surrounding attitudes towards antibiotic use.

Six hundred (85.5%) participants agreed that pregnant women need to consult a doctor before taking antibiotics. However, only 75.2% agreed that pregnant women should not buy any antibiotics over the counter. Also, 55.6% agreed it is not good to accept antibiotics from family members to treat infection (Figure 2).

### 3.4 Practices of pregnant women regarding antibiotics

**Figure 3:**
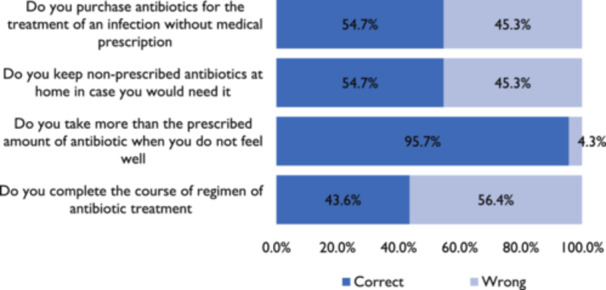
Percentage of responses from all respondents to statements to determine practice towards antibiotic use.

In this study, 45.3% of the women reported having engaged in self‐medication through the purchase of unprescribed antibiotics over the counter whiles pregnant. Only 43.6%, completed the course of antibiotic regimen for treatment and 54.7% do not keep antibiotics at home (Figure 3).

### 3.5 Predictors of knowledge of antibiotic use and resistance among pregnant women

To identify the predictors of knowledge among pregnant women, we performed a logistic regression analysis to compare participants' demography and knowledge score (Table 2.) The regression model analysis reveals that pregnant women who have had primary secondary and tertiary education were 16.61 (CI: 9.523–29.46; *p* < 0.001), 21.27 (CI: 12.19–37.45; *p* < 0.001), and 34.67 (CI: 16.99–71.94; *p* < 0.001) times likely to be knowledgeable than those who had never had formal education. Also, we found out that increased monthly income is associated with an increased likelihood to have good knowledge as those who earned between Gh¢500–999, Gh¢1000–1499, Gh¢1500–1999, and Gh¢2000 or more were 3.429 (CI: 2.257–5.186; *p* < 0.001), 4.571 (CI: 2.588–7.962; *p* < 0.001), 8.571 (5.112–14.06; *p* < 0.001), and 14.86 (7.594 to 28.22; *p* < 0.001) times likely to have good knowledge that those that earn <Gh¢500. We also detected that good knowledge is associated with a 40% decrease in the odds of keeping nonprescribed drugs at home.

**Table 2:** Logistic regression analysis of predictors of knowledge among study participants.

Characteristics	OR	95% CI	*p*
Marital status			
Not married	1		
Married	1.722	0.9324–3.163	0.109
Educational Level			
None	1		
Primary	16.61	9.523–29.46	<0.001
Secondary	21.27	12.19–37.45	<0.001
Tertiary	34.67	16.99–71.94	<0.001
Monthly income			
<500	1		
500–999	3.429	2.257–5.186	<0.001
1000–1499	4.571	2.588–7.962	<0.001
1500–1999	8.571	5.112–14.06	<0.001
≥2000	14.86	7.594–28.22	<0.001
Pregnant women should not buy antibiotics over the counter			
Disagree	1		
Agree	1.444	0.9614–2.170	0.088
Neutral	0.5714	0.3008–1.093	0.086
Do you keep non‐prescribed antibiotics at home in case you would need it			
No	1		
Yes	1.407	1.039–1.888	0.027
Do you purchase antibiotics from over‐the‐counter for an infection?			
No	1		
Yes	7.554	5.366–10.55	<0.001

Abbreviations: 95% CI, 95% confidence interval; OR, odds ratio.

### 3.6 Predictors of antibiotic self‐medication in pregnant women

As indicated in Table 3, education, monthly income, good practice, and good knowledge were significantly associated with a reduced likelihood to self‐medicate. Pregnant women who have had primary, secondary and tertiary had 0.2679 (CI: 0.1707–0.4152; *p* < 0.001), 0.1296 (CI: 0.08330–0.2039; *p* < 0.001), and 0.1157 (CI: 0.06585–0.2070; *p* < 0.001) reduced odd of self‐medicating with antibiotics. Also, we found out that an increase in monthly income is associated with decreased odds of self‐medication. Those who earned Gh¢1000–1499, Gh¢1500–1999, and Gh¢2000, or more had reduced odds of 0.05329 (CI: 0.02415–0.1279; *p* < 0.001), 0.1563 (CI: 0.09444–0.2591; *p* < 0.001), 0.04187 (CI: 0.01918–0.09848; *p* < 0.001), respectively. We realized that desired practice of completing the course of the regimen of antibiotics for treatment was associated with a 58.4% decrease in the odds of self‐medication with antibiotics. Those who keep unprescribed antibiotics at home were 2.69 (CI: 1.989–3.672; *p* < 0.001) times more likely to engage in self‐medication with antibiotics. Those who had good knowledge were associated with an 86.8% decrease in odds of self‐medication.

**Table 3:** Logistic regression analysis of predictors of antibiotic self‐medication among study participants.

Characteristics	OR	95% CI	*p*
Age (years)			
16–20	1		
21–25	0.5752	0.3529–0.9587	0.032
26–30	0.5625	0.3468–0.9126	0.025
31–35	0.5625	0.3090–0.9945	0.057
36–40	1.607	0.9246–2.838	0.123
41–45	0.375	0.1397–1.042	0.050
Educational level			
None	1		
Primary	0.2679	0.1707–0.4152	<0.001
Secondary	0.1296	0.08330–0.2039	<0.001
Tertiary	0.1157	0.06585–0.2070	<0.001
Monthly Income			
<500	1		
500–999	1.507	0.9870–2.322	0.069
1000–1499	0.05329	0.02415–0.1279	<0.001
1500–1999	0.1563	0.09444–0.2591	<0.001
≥2000	0.04187	0.01918–0.09848	<0.001
Gravidity			
First	1		
Second	1.436	0.8871–2.281	0.154
Third	0.8889	0.5546–1.401	0.638
Fourth	1.556	0.9807–2.494	0.073
Fifth or more	0.5926	0.3308–1.060	0.103
Parity			
Nulliparous	1		
Primiparous	1.296	0.8246–2.010	0.249
Multiparous	1.502	1.014–2.267	0.047
Grand multiparous	0.5185	0.1980–1.384	0.186
How many lost pregnancy			
None	1		
One	1.55	1.111–2.140	0.008
Two	1.387	0.6979–2.754	0.344
Three	0.2312	0.02003–1.418	0.141
Pregnant women should not accept antibiotics from a family member for treatment of an infection			
Disagree	1		
Agree	0.531	0.3835–0.7304	<0.001
Neutral	0.2125	0.1098–0.4059	<0.001
Pregnant women should not buy antibiotics over the counter			
Disagree	1		
Agree	0.5804	0.3823–0.8894	0.010
Neutral	0.96	0.5319–1.747	0.900
Do you complete the course of regimen of antibiotic treatment			
No	1		
Yes	0.4167	0.3074–0.5659	<0.001
Do you keep nonprescribed antibiotics at home in case you would need it			
No	1		
Yes	2.69	1.989–3.672	<0.001
Knowledge score			
Poor knowledge	1		
Good knowledge	0.1324	0.09478–0.1864	<0.001

Abbreviations: 95% CI, 95% confidence interval; OR, odds ratio.

## 4 DISCUSSION

This study was conducted to assess the KAP of antibiotic use and the prevalence of antibiotic self‐medication among pregnant women receiving ANC in a primary health facility in Northern Ghana.

In this study, 55.6% of pregnant women had good knowledge of antibiotic use and resistance, which is however lower than the 80% reported among pregnant women in South Africa.^15^ Studies in different parts of the world indicate different levels of self‐medication among pregnant women.^8^, ^17^, ^24^, ^25^, ^26^ In this study, 45.3% of the women reported having purchased antibiotics over the counter without a prescription while pregnant. This rate is in contrast to the 16.6% in South Africa,^15^ 12.5%, and 25.1% in Ethiopia,^27^, ^28^ and 37% in Nigeria^9^ that has been reported among pregnant women. Reasons attributed to self‐medication include the urge for self‐care, feeling of sympathy toward family members in sickness, lack of time, lack of health services, financial constraint, ignorance, misbelieves, extensive advertisement, and availability of drugs in informal channels are responsible for the growing trend of self‐medication.^8^, ^29^

The antibiotic resistance crisis is not only a health threat to the healthcare industry but also an economic burden for both developed and developing economies. The consequences of antibiotic resistance on patient care is dire and cannot be estimated especially in resource limited countries including Ghana.^11^, ^30^ A major reason responsible for the antibiotic resistance crisis is overdosage and self‐medication.^30^ Self‐medication is influenced by many factors.^4^, ^17^, ^18^, ^31^, ^32^, ^33^ In this study, we report that high education level, monthly income, good practice, and good knowledge were significantly associated with a reduced likelihood to self‐medicate. Pregnant women who have had primary, secondary, and tertiary had reduced odds of self‐medicating with antibiotics. In a study conducted in Lebanon by Jamhour et al.,^34^ self‐medication significantly correlated with a lower educational level. This may be because better medication knowledge is associated with higher educational levels^35^ and also increase the odds of maintaining excellent or very good health.^36^

Also, we found out that an increased monthly income is associated with decreased odds of self‐medication. Those who earned Gh¢1000–1499, Gh¢1500–1999, and Gh¢2000 or more had reduced odds of self‐medicating, in contrast to KAP studies among the South African cohort of pregnant women where women with higher purchasing power were more likely to self‐medicate.^15^ This may be because high earners can afford the cost of medical care and the cost of drugs that can be purchased with prescription.^37^, ^38^

We realized pregnant women who had good knowledge of antibiotics completed the course of the regimen of antibiotics for treatment and had reduced odds of practicing self‐medication with antibiotics. A similar situation was detected by Jamhour et al.,^34^ where those with poor knowledge about antibiotics stopped antibiotics at the inappropriate time and were involved in self‐medication. Also, a study in South Africa reported that participants with good knowledge were six times more likely to have good practice regarding antibiotic use.^15^ Pregnant women who had poor knowledge kept unprescribed antibiotics at home and were more likely to engage in self‐medication with antibiotics. Such poor practices regarding antibiotic use have been reported in many countries in Africa^3^, ^9^, ^12^, ^15^, ^16^, ^25^, ^27^, ^38^, ^39^ and Europe.^21^, ^24^

The lack of knowledge about self‐medication among the study participant may be contributing to the antibiotic resistance tragedy.^3^ There is therefore the need for community education about antibiotics, their use, and side effects, and to discourage them from self‐medication and misuse of antibiotics^3^ as the Antibiotic resistance crisis is not just a concern related to the health care industry, but can impact a country's economic growth as well.^40^

## 5 CONCLUSION

Even though 55.6% of the pregnant women demonstrated good knowledge, the prevalence of self‐medication is significantly high. We report that high education level, monthly income, good practice, and good knowledge were significantly associated with a reduced likelihood of self‐medication with antibiotics. An increase in knowledge among pregnant women about antibiotics has the potential of reducing the odds of antibiotic self‐medication by 87%. The results of this study underscore the need for effective measures and interventions to discourage self‐medication among this group. A well‐structured education that could be easily accepted and understood by pregnant women on the risks of antibiotic self‐medication should be included in the routine education at the antenatal clinics.

## AUTHOR CONTRIBUTIONS

Ezekiel K. Vicar contributed to study conceptualization, design, data analysis, drafting, and revision of the manuscript. Williams Walana contributed to study conceptualization, data analysis, drafting and revision of the manuscript. Rosemond A. Fordjour contributed to methodology, investigation, drafting, and revision of the manuscript. Christiana Benneh contributed to methodology, investigation, drafting, and revision of the manuscript. Rosemond E. Bentil contributed to study design, data collection, analysis, and revision of the manuscript. Gifty M. Wuffelle contributed to the conceptualization, study supervision, and revision of the manuscript. Emmanuel K. Osabutey contributed to formal analysis, supervision, and revision of the manuscript. Gilbert Nachinab contributed to the data analysis, interpretation, drafting and revision of the manuscript. Mauvina Obeng‐Bempong contributed to study conceptualization, design, drafting and revision of the manuscript. All authors read and approved the final draft of the manuscript.

## CONFLICT OF INTEREST STATEMENT

The authors declare no conflict of interest.

## TRANSPARENCY STATEMENT

The lead author Ezekiel K. Vicar affirms that this manuscript is an honest, accurate, and transparent account of the study being reported; that no important aspects of the study have been omitted; and that any discrepancies from the study as planned (and, if relevant, registered) have been explained.

## Data Availability

General data from this study will be made available upon request to the corresponding author.
